# Quicksilver & Gold: Mercury Pollution from Artisanal and Small-Scale Gold Mining

**DOI:** 10.1289/ehp.120-a424

**Published:** 2012-11-01

**Authors:** Charles W. Schmidt

**Affiliations:** **Charles W. Schmidt**, MS, an award-winning science writer from Portland, ME, has written for *Discover Magazine*, *Science*, and *Nature Medicine*.


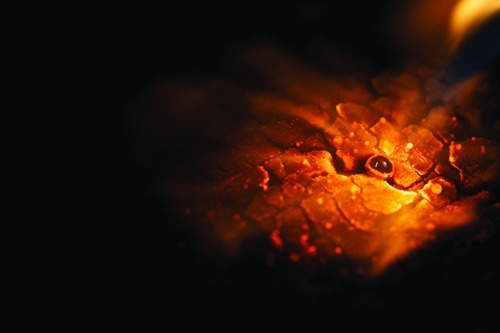
In 2009 a mining engineer named Marcello Veiga set out to study mercury air pollution in a part of northwest Colombia called Antioquia Department. This mountainous, conflict-ridden state, where leftist guerillas routinely battle Colombian security forces, is an important center for artisanal and small-scale gold mining (ASGM). Miners isolate gold by mixing ores dug from the ground or from stream beds with mercury to form an amalgam. When the amalgam is burned, the elemental mercury vaporizes into a toxic plume while the gold stays behind.

Veiga, who is also an associate professor at the University of British Columbia, was especially interested in air quality in and around specialized shops called *entables* that burn amalgam for a fee. Faced with threats of robbery in the field, local miners bring their ore to these *entables* for final processing. Five cities in Antioquia—Segovia, Remedios, Zaragoza, El Bagre, and Nechi—collectively house more than 300 *entables*, each of them a point source for inorganic mercury vapors that pollute the air of the region and beyond.

Antioquia’s *entables* produce 10–20 metric tons of pure gold each year, so Veiga expected the air mercury levels would be high. Still, he was surprised when readings occasionally spiked over 999 µg/m^3^, the upper limit on his handheld mercury analyzer.^1^ That’s nearly 1,000 times the World Health Organization’s (WHO) air quality guideline of 1 µg/m^3^ for chronic exposure to inorganic mercury vapor^2^ and 3,000 times the U.S. Environmental Protection Agency reference concentration of 0.3 µg/m^3^ for chronic inhalational exposure to inorganic mercury.^3^

**Figure:**
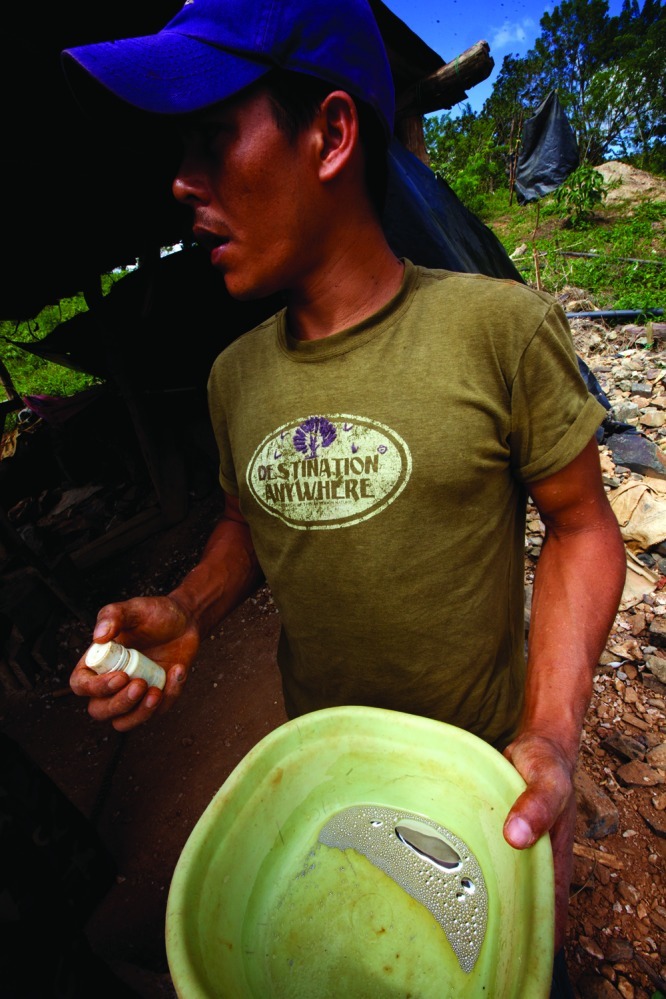
For centuries miners have used mercury to trap particles of precious metals. Artisanal and small-scale gold miners used an estimated 1,400 metric tons of mercury in 2011. About one-third of the mercury used is believed to go into the air while the rest goes into soils and waterways. All images © 2012 Sean Hawkey

The worst pollution was in the *entables*, even on their days off. But Veiga also detected inorganic mercury levels tens to hundreds of times higher than the WHO air quality guideline in city plazas, local neighborhoods, a bakery, and in front of an elementary school in Remedios, where readings ranged from 5 to 10 µg/m^3^. “These were the highest mercury levels I’d ever seen,” says Veiga, whose research experience in gold mining spans three decades in 40 countries.

Mercury amalgamation has been used for centuries to process precious metals.^4^ Today, ASGM is the world’s second greatest source of atmospheric mercury pollution after coal combustion, according to the United Nations Environment Programme (UNEP).^5^ And with gold prices now exceeding US$1,600 per ounce (up from less than US$500 in the 1980s),^6^ ASGM is on the rise along with its mercury problem, Veiga says.

## A Persistent Problem

More mercury is used for ASGM—an estimated 1,400 metric tons in 2011—than for any other use of the metal.^5^ The ASGM industry emits an estimated annual average of 1,000 metric tons of inorganic mercury, about one-third of which is thought to go into the air while the rest winds up in piles of mining waste (“tailings”), soils, and waterways.^7^ Some of the inorganic mercury that reaches aquatic ecosystems also gets converted by microbes into organic methylmercury, which accumulates in fish.

Both the inorganic and organic forms can cause neurological problems. However, methylmercury, which passes more easily into the brain, is generally considered the more toxic species, particularly among children, who can experience IQ losses, delayed speech, and other neurodevelopmental deficits from exposure. Early-life exposures are the most harmful, says Roberta F. White, chair of environmental health and associate dean for research at the Boston University School of Public Health, because they can damage the whole brain. Exposures later in life, on the other hand, produce more localized damage to the cerebellum, visual cortex, and motor strip. In adults, these exposures can lead to visuospatial problems and effects on executive functioning, memory, and mood.^8^

Some 10–15 million people in 70 countries work in the ASGM trade. The estimated number of child workers varies; in the African Sahel, for instance, they could make up 30–50% of the workforce^9^ while mining operations in the Brazilian Amazon employ far fewer children, according to Veiga.

Last year, investigators with the New York–based nongovernmental organization Human Rights Watch (HRW) traveled to Mali, where children as young as 6 were seen digging mine shafts, carrying and crushing stone, and panning for gold alongside adults. In a December 2011 report, the group claims that 20,000–40,000 children from that country work in gold mining, and that many among them carry out amalgamation, which can result in protracted exposure to mercury vapor.^10^ “And virtually none of the children knew that mercury is toxic,” says Juliane Kippenberg, an HRW senior researcher. “Very few were taking precautions against the fumes.”

**Figure:**
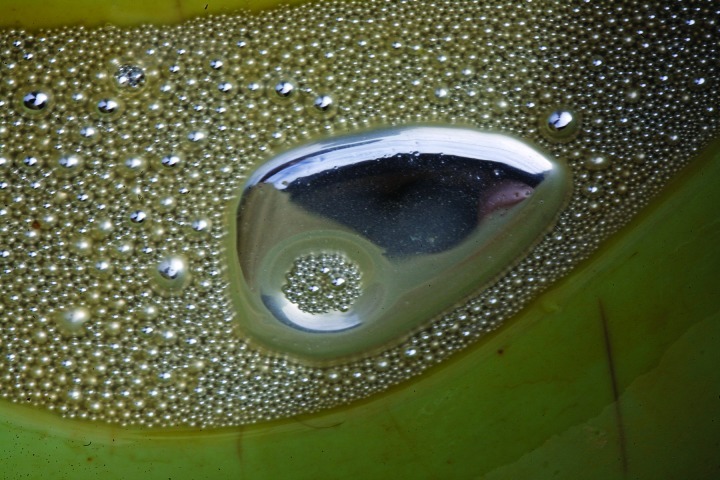
Why Is Mercury Used? Mercury offers several benefits in gold processing: It is easy to use, and it works quickly.It can be used by one person independently.It effectively extracts gold in most field conditions.It is cheaper than most alternative techniquesIt facilitates precise transactions.It permits custom processing of small individual ore batches. Miners often are not aware of the risks involved in using mercury, and/or they may not have a choice in the matter. Those miners who are aware often do not afford or have access to safer alternatives. Telmer and Stapper (2012)^5^

In his field work Veiga routinely sees pregnant women and women of childbearing age burning amalgam, sometimes because the men refuse to. “I’ve spoken with male miners in Sudan who claim that ‘only women can do the delicate work of amalgamating gold,’ ” he says.

But the mercury from ASGM operations travels beyond the job site, exposing not only miners but also their families to elemental mercury that spreads through the air and soil in mining communities. In addition, mining pollution in aquatic ecosystems can expose downstream communities to methyl-mercury through their diet.

Mercury from ASGM operations can also travel in the atmosphere to fall out with deposition thousands of miles from its source, according to Donna Mergler, a professor emerita at the University of Québec in Montréal. “So the pollution becomes a worldwide phenomenon,” she says. And that, says Susan Keane, a senior environmental analyst with the Natural Resources Defense Council, is why mercury pollution from ASGM cannot simply be considered a local problem:  “It is part of the bigger global mercury pollution story,” she says.

## Investigating Toxicity and Environmental Fate

Health studies of ASGM communities are rare and challenging to carry out because other confounding factors can influence neurological performance. Few researchers have devoted more effort to these studies than Stephan Böse-O’Reilly, a pediatrician and epidemiologist at the University of Munich.

In 2008 Böse-O’Reilly published a study of 166 children, aged 9–17, from ASGM areas in Zimbabwe, Tanzania, and Indonesia.^11^ He found that those with the highest exposures had symptoms of mercury poisoning, such as excessive salivation, a metallic taste in the mouth, and abnormal reflexes. In addition, elevated mercury exposure was linked to lesser performance on two neuro-logical assays: the matchbox test, which meas-ures how long it takes to put 20 matches into a box using alternating hands, and the pencil tapping test, which measures how many dots children can tap onto a piece of paper in 10 seconds.^10^ “These results indicate ataxia, or coordination problems resulting from damage to the cerebellum,” Böse-O’Reilly explains.

White and colleagues also linked mercury exposure to neurotoxicity in children living near ASGM sites in northern Brazil. They assessed a total of 351 children, aged 7–12 years, from villages on the Tapajós River, an Amazon tributary. More than 80% of the children had hair mercury levels of at least 10 µg/g, over which neurotoxicity can be expected, White says. Tests of motor function, attention, and visuospatial function also showed decrements in performance with increasing exposure.^12^

However, in reporting these findings, the investigators pointed out that confounding effects from tropical disease or prior nutritional deficiencies may have influenced the results. Moreover, another neurological study of children living near ASGM sites in French Guyana, conducted by a team including some of the same investigators, produced less conclusive findings, even though the hair mercury levels were higher, averaging 12.7 µg/g among both mothers and their children. That study detected no major neurological problems, although dose-dependent relationships were noted between the amounts of mercury in maternal hair and poor leg coordination, and reduced measures of visuospatial performance in male children.^13^

**Figure:**
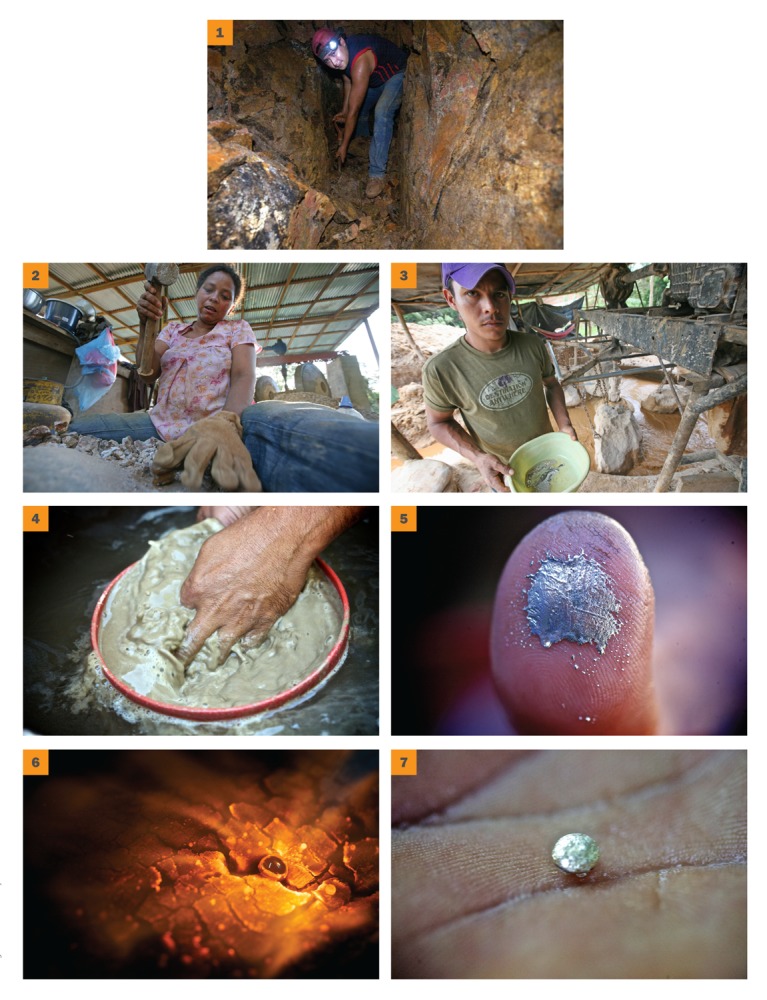
**Steps in Small-Scale Gold Mining** 1) Miners excavate gold ore from the ground or from rivers. 2) Ore is broken aboveground and tested for gold content. 3) Broken ore is put into the mills. Mercury is added to the mill to bind the tiny gold particles together. The mercury–gold amalgam is heavier than crushed rock particles and sinks to the bottom of the mill. Gold particles that don’t come into contact with mercury in the mill can be caught on a mercury-covered plate at the runoff. 4) The muddy mixture of rock particles that is left in the bottom of the mill is washed and the amalgam separated out. 5) Excess mercury is either squeezed out of the amalgam by hand (which saves the mercury for further use) or burned off. 6) Amalgam is burned with a blowtorch to burn off the mercury. 7) The resulting low-purity “doré” gold is sent to refineries to be further purified. All images © 2012 Sean Hawkey

A 2009 review article of hair mercury levels in the Amazon emphasized that their association with neurological abnormalities in the region aren’t easily identifiable.^14^ According to White, that’s in part because differences in culture, education, and background health among populations can influence neuropsychological testing. “In French Guyana, education and endemic malaria were both problems,” she says.

Meanwhile, the degree to which ASGM contributes to mercury contamination in the Amazon remains unclear. The Tapajós River study showed that hair mercury levels rise with increasing proximity to the mining sites, suggesting that tailings constitute the primary exposure source. However, ASGM sites are not the only source of mercury in the Amazon—given their volcanic origins, Amazonian soils tend to be naturally high in mercury, Mergler explains, and much of what’s found in fish and the river comes from soil erosion triggered by deforestation.^15^ But she adds that “increased mercury in water and fish is not ‘natural.’ The current increase in deforestation in the Brazilian Amazon and more particularly along the Tapajós River has serious consequences for human health and the environment.”

Rebecca Adler Miserendino, a PhD candidate at the Johns Hopkins Bloomberg School of Public Health, says questions about the sources and movements of mercury in the Amazonian environment persist in part because sediment and soil samples tend to be minimally characterized. In work that has been submitted for publication, Adler-Miserendino says she provides data that could help to resolve questions about anthropogenic versus natural mercury contributions in South America. She declined to comment on the data but did emphasize that mercury movements near ASGM sites remain understudied. And findings from the Amazon, she cautions, can’t necessarily be extrapolated to other regions in the world where geochemical and mining conditions may differ, including Africa, where these types of studies have not been performed.

## Confronting the Problem

ASGM varies widely in terms of its scope and practice. In some cases, villagers find gold serendipitously, or by trial and error, triggering an influx of miners who come by the thousands and then leave when the source runs dry. Other areas, such as those in the Amazon River Basin, have more established mines with operational histories that can date back decades. Some countries ban mercury use in ASGM outright, says Keane. In other cases, it’s the mining itself that’s illegal, because the workers who do it don’t have ownership or mineral rights to the land. But enforcement is lax in poor countries, and apart from isolated incidents in which police round up miners in a show of authority, governments rarely intervene, Veiga says.

**Figure:**
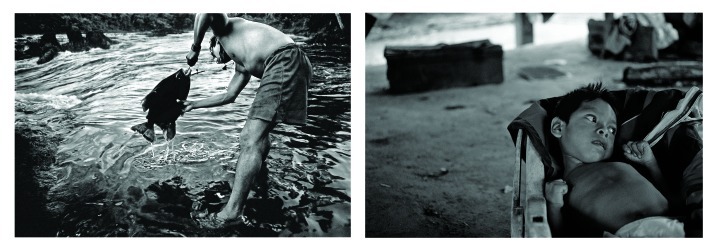
Inorganic mercury makes its way into soils and waterways where anaerobic bacteria convert it to highly toxic methylmercury. Methylmercury accumulates in aquatic animals, including the fish that are a staple of many Amazonian peoples’ diet. Exposure to methylmercury is widespread and often severe enough to result in adverse neurological effects. © 2012 Christophe Gin/Corbis

According to Sam Spiegel, a lecturer in international development at the University of Edinburgh, efforts to lower mercury emissions should aim to support miners and their livelihoods by supplying access to better technology. “This doesn’t mean eliminating mercury right away,” Spiegel says. “It means coming up with ways to use it more efficiently.”

Policy makers with UNEP agree. In a practical guide for how to reduce mercury uses in ASGM published in 2011, UNEP recommends a two-step approach: start by limiting mercury uses with improved practices, then move toward mercury-free technologies that either boost or maintain miner income while protecting health and the environment.^5^

UNEP and other sources interviewed for this story single out “whole-ore amalgamation” as a major problem. This practice, which entails adding mercury to ore as it comes out of the ground, generates vast amounts of toxic waste. Whole-ore amalgams rarely capture more than a third of the gold^5^ and lose the rest—along with the mercury—in tailings. According to Veiga, some *entables* in South America leach residual gold from tailings using cyanide, producing a waste he calls a “toxic bomb” that’s often discharged directly into the environment.

But by concentrating ores with basic separators or more expensive industrial centrifuges, miners can reduce the amount of mercury needed for amalgamation by up to 90%, Veiga says. With funding from the United Nations Industrial Development Organization (UNIDO), the Global Mercury Partnership—a voluntary collaboration of government, nongovernment, public, and private entities—has trained miners how to build these devices using readily available materials. Low-tech gravimetric devices, for instance, pass liquid slurries of water and crushed ore over a fabric surface that traps gold-bearing particles.

“We work with a lot of transient communities,” says Ludovic Bernaudat, an industrial development officer with UNIDO. “If we can teach them how to generate more gold with less waste, they take that knowledge with them. We can’t train fifteen million people, but those we do train expand our reach.”

Miners can also use “retorts,” or simple fume hoods that keep mercury out of the air during amalgamation. According to UNEP’s guidance document, retorts that heat amalgam and then condense mercury vapor back into a liquid form can cut toxic air emissions by 75–95%. However, UNEP warns that children and women of childbearing age should not use retorts or be present during retorting.^5^

## Global Forces

Market forces have also begun to play a role in cleaning up ASGM. The Fairtrade Foundation, in London, United Kingdom, for instance, has a Fairtrade and Fairmined program that certifies gold that’s been extracted using safe and responsible practices for managing mercury and other toxics.^16^ Chemical use is also minimized, but many Fairtrade miners work in areas where the type of gold deposit, plus the geography and available resources, mean they have no alternative to using mercury and other toxic chemicals to extract the gold they produce, according to Gemma Cartwright, the program’s coordinator. Certified workers must also use personal protective equipment. In addition, mines may not employ any workers under age 15, and workers under 18 may not work in hazardous conditions.

UNEP will soon wrap up a legally binding global treaty on mercury, including a section on ASGM.^17^ At the fourth session of the intergovernmental negotiating committee, which convened in Punta del Este, Uruguay, in June 2012, delegates drafted language that calls on parties to reduce and (where feasible) eliminate the use of mercury in mining and gold processing.^17^

The language also states that parties have an obligation to develop national action plans for reducing exposure to vulnerable populations, in particular children and pregnant women, and for taking steps to limit the worst practices, such as burning amalgam in residential areas. “We’re happy to know that things have gotten that far and that the national plans will be mandatory,” Kippenberg says. “However, we are missing a clear prohibition of children’s use of mercury in the treaty.”

But to HRW’s dismay, the U.S., Canadian, and European delegates were reluctant to adopt a separate article on health proposed by Latin American and Caribbean countries, which calls for monitoring to identify all populations at greatest risk from mercury exposure, not just those working in ASGM. The measure also states that parties shall “facilitate and assure proper access to health care for populations affected by the exposure to mercury or its compounds.”^18^

U.S. delegates refused to comment on the treaty while negotiations are ongoing. But according to Kippenberg, the delegates claim the measure would compel developed countries to fund health care programs that divert resources away from reducing environmental mercury exposure. The treaty is expected to be finalized by January 2013.

Ultimately, efforts to confront mercury pollution from ASGM must also consider the trade’s role in alleviating poverty for millions of people, Spiegel says. For many of them, gold mining wouldn’t be financially or technically feasible in the short term without mercury, creating a real quandary for how to balance environmental protection with economic development.

“The important thing is that you don’t blame the miners,” says Böse-O’Reilly. “The solution isn’t that they stop mining but that they reduce and then replace the mercury with something else, which is good for all of us. You don’t want these people to lose jobs that they urgently need.”
